# Supervised (Home-Based Exercise) Prehabilitation Program in Pancreatic Cancer Patients Undergoing to Neoadjuvant Chemotherapy: A Pilot Feasibility Study

**DOI:** 10.3390/medsci14020184

**Published:** 2026-04-07

**Authors:** Gennaro Boccia, Luca Beratto, Cantor Tarperi, Alberto Rainoldi, Chiara Calliera, Daniele Ierace, Maria Antonietta Satolli, Simona Bo, Paola Costelli

**Affiliations:** 1 Department of Clinical and Biological Sciences, University of Torino, 10043 Orbassano, Italy; gennaro.boccia@unito.it; 2 Department of Medical Sciences, University of Torino, 10126 Torino, Italy; luca.beratto@unito.it (L.B.); alberto.rainoldi@unito.it (A.R.); simona.bo@unito.it (S.B.); 3 Department of Neurosciences, Biomedicine and Movement Sciences, University of Verona, 37124 Verona, Italy; cantor.tarperi@univr.it; 4 Department of Anesthesiology and Critical Care, AOU Città della Salute e della Scienza, 10126 Torino, Italy; chiara.calliera@gmail.com; 5 Department of Oncology, University of Torino, 10126 Torino, Italy; daniele.ierace@unito.it (D.I.); mariaantonietta.satolli@unito.it (M.A.S.)

**Keywords:** pancreatic cancer, prehabilitation, feasibility, home-based exercise, HIIT, cardiopulmonary exercise testing

## Abstract

Background: Patients with pancreatic cancer (PC) commonly present with reduced aerobic fitness, sarcopenia, and malnutrition, which may increase perioperative risk and compromise access to chemotherapy treatments. Although exercise-based prehabilitation can improve physical fitness, its implementation is often limited by short diagnostic-to-surgery intervals and treatment-related toxicity. Methods: We conducted a pilot prospective pretest–posttest feasibility study in Torino, Italy. Patients with PC undergoing neoadjuvant chemotherapy prior to surgery were offered a 4-week, partially supervised, home-based bimodal exercise prehabilitation program (single-arm design) combining remotely monitored high-intensity interval training (HIIT) on a cycle ergometer with functional and resistance exercises. The primary outcome was adherence to prescribed exercise frequency, intensity, and duration, objectively assessed via remote monitoring. Secondary outcomes included cardiorespiratory fitness (CPET), muscle function, body composition, fatigue, quality of life, and circulating inflammatory markers. Results: From July 2022 to February 2024, 23 patients were screened; 15 were eligible and 10 enrolled. Four participants discontinued the intervention (two due to asthenia/fatigue, one due to chemotherapy-related adverse events, and one for organizational reasons), leaving six participants who completed the program. Among completers, fatigue and quality of life did not change meaningfully. Aerobic capacity and muscle function outcomes were generally stable, with few pre–post changes exceeding the minimum clinically important difference (MCID) thresholds used. Body composition markers and the assessed circulating cytokines/chemokines remained unchanged except for IL-6 levels, which decreased significantly (*p* < 0.05). Conclusions: A partially supervised, home-based HIIT-based prehabilitation program is feasible for a subset of PC patients undergoing neoadjuvant therapy, but a substantial attrition rate suggests the need for more flexible symptom-adapted prescriptions and enhanced supportive strategies.

## 1. Introduction

Patients affected by pancreatic cancer show a 5-year survival rate below 13%. This type of tumor is foreseen to become the second cause of death for cancer by 2030 [1]. Besides being among the most aggressive tumors, pancreatic cancer is frequently diagnosed when the disease stage is advanced, seriously challenging the effectiveness or even the possibility to perform a radical surgical resection: about 15 to 20 percent of all pancreatic tumors are resectable [2]. Potentially curative surgeries are procedures that aim for “complete resection” and are reserved for localized and not metastatic cancer. Pancreato-duodenectomy (or the Whipple procedure) is the most common surgery for head pancreas tumors. It removes the pancreatic head, duodenum, gallbladder, part of the bile duct, and often a portion of the stomach. A variation in the Whipple procedure keeps the pylorus valve (Pylorus-Preserving Whipple). Distal pancreatectomy is performed for body or tail pancreas tumors. And the spleen is also frequently removed during this procedure. Total pancreatectomy removes the entire pancreas with the gallbladder, part of the stomach and small intestine, and the spleen (https://cancer.org). At present, this latter procedure is the only potentially curative treatment option, while chemotherapy-based approaches, offered to patients with advanced, non-operable cancers, rapidly lose their efficacy due to the development of chemoresistance [3]. Systemic chemotherapy is the initial treatment strategy for borderline resectable and locally advanced pancreatic cancer to facilitate curative resection [4].

In addition to late diagnosis, patient fitness is one of the most relevant issues undermining the surgical option [5,6]. Indeed, the occurrence of malnutrition, obstructive jaundice, sarcopenia and low aerobic fitness eventually result in a reduced capacity to cope with surgery-related stress [7,8,9]. As a consequence, the likelihood of postoperative complications in these patients is high [10,11]; this risk is further increased in patients undergoing neoadjuvant chemotherapy [12]. Previous observations showed that pre-surgery aerobic fitness correlates well with post-surgery complications; for example, patients with an Gas Exchange Threshold (GET) below 10 mL/kg/min showed higher rates of postoperative complications and were less likely to receive adjuvant therapy [11,13,14]. Therefore, patient stratification according to their fitness will help to identify those who could benefit from a prehabilitation intervention aimed at reducing the perioperative risk. Specific prehabilitation programs (preceding surgery or chemotherapy) have been developed or are under investigation [15,16]. Most of them are based on combinations of physical activity, nutritional interventions and psychological support [17].

Physical training has been shown to effectively influence aerobic fitness, assessed as O_2_ uptake (V̇O_2_) at the GET and V̇O_2_ at peak exercise (V̇O_2peak_) [18,19]. Pre-surgery aerobic capacity was improved in patients enrolled in a 6-week prehabilitation program mainly based on endurance exercise training. Post-surgery complications were reduced in the same patients [20]. Physical prehabilitation both in-hospital (supervised) and at home (unsupervised) determined increased physical fitness but no improvement of post-surgery complications [21].

It is now well accepted that patients prefer a home-based approach [22,23], since patients could comply better with this approach than to an in-hospital program. Home-based protocols, however, should be supervised to ensure a true compliance [8]. Nevertheless, the available time-window to set up a prehabilitation program before surgery usually does not exceed 4–6 weeks. The high-intensity interval training (HIIT) appears the most effective one for this short time interval [24,25]. Four weeks of prehabilitation including HIIT was well tolerated by patients scheduled for pancreatic surgical resection, resulting in an improved aerobic capacity [24]. However, the feasibility and acceptability of HIIT in this patient population remain insufficiently characterized.

The present study aimed to investigate whether a prehabilitation program consisting of 4 weeks of partially supervised home-based exercise was feasible in patients with borderline resectable or locally advanced pancreatic cancer scheduled to undergo neoadjuvant chemotherapy with subsequent evaluation for possible pancreatic resection. We hypothesized that a 4-week partially supervised home-based exercise prehabilitation program combining remotely monitored HIIT with functional/resistance exercises would be feasible in patients with pancreatic cancer undergoing neoadjuvant chemotherapy. Specifically, we expected acceptable participation, retention, and adherence, together with a low incidence of exercise-related adverse events. In addition, data on aerobic capacity, muscle mass and function, fatigue and quality of life were assessed.

## 2. Materials and Methods

### 2.1. Study Design

This was a pilot feasibility study employing a single-arm pre–post test design. The protocol was approved by the local Ethical Committee (N. 102/2022) and was in line with the Declaration of Helsinki. All patients gave their written informed consent to participate. This study was conducted within the broader framework of a multicenter protocol (ClinicalTrials.gov ID NCT05496777; registered 11 August 2022), but the present manuscript reports a center-specific exploratory cohort recruited in Torino, Italy. This decision was made because the Italian cohort differed clinically from the cohort described in the registered multicenter protocol and associated publications [26,27]. Specifically, our center enrolled patients with borderline resectable (BRPC) or locally advanced (LAPC) pancreatic cancer undergoing neoadjuvant chemotherapy before possible surgery, whereas the other participating center focused on unfit patients already scheduled for pancreatic surgery [27]. Given these differences in clinical pathway, timing, and eligibility context, the present data are reported separately and should be interpreted as a pilot feasibility analysis of a distinct exploratory cohort.

### 2.2. Eligibility/Exclusion Criteria

Participants were eligible if they met all of the following criteria: (1) age ≥ 18 years; (2) diagnosis of borderline resectable or locally advanced pancreatic cancer and scheduled to undergo neoadjuvant chemotherapy with subsequent evaluation for possible pancreatic resection; (3) willing to participate in the home-based prehabilitation program; (4) provided informed consent to participate. Exclusion criteria included: (1) the need for acute surgery; (2) scheduled surgery at another hospital; (3) inability to perform cycling exercise; (4) medical contraindications to physical training (e.g., cardiac arrhythmias, myocardial ischemia); (5) inability to comply with testing procedures (e.g., insufficient understanding of the Italian language); (6) unavailability of a certified kinesiologist in the patient’s area of residence.

### 2.3. Recruitment

Eligible patients were identified during Multi-Disciplinary Team (MDT) meetings. Patients received detailed verbal and written information about the study during the next medical oncology outpatient visit. Those expressing interest were scheduled, and written informed consent was obtained.

No formal a priori sample size calculation for efficacy was performed because the primary aim of this study was to assess feasibility rather than to test the effectiveness of the intervention on clinical or physiological outcomes. In accordance with the design of an early-stage feasibility study, the sample size was pragmatically determined by the number of eligible patients who could be recruited within the study period at our center and who met the logistical requirements for participation in the home-based supervised program.

### 2.4. Study Outcomes

The primary objective of this study was to assess the feasibility of a four-week partially supervised home-based prehabilitation program in patients with pancreatic cancer undergoing neoadjuvant chemotherapy before possible pancreatic resection. Feasibility was evaluated through participation rate, retention/drop-out, adherence to prescribed training frequency, intensity, and duration, and the occurrence of exercise-related adverse events. Secondary objectives were exploratory and included cardiorespiratory fitness, muscle function, functional activities, skeletal muscle mass and quality, cytokine levels, fatigue, and health-related quality of life.

### 2.5. Intervention

The exercise prehabilitation program consisted of three sessions per week over four weeks. An experienced exercise professional visited participants at home to provide equipment instruction and to supervise sessions (three supervised sessions during week 1 and one supervised session per week during weeks 2–4). Each supervised session included a Steep Ramp Test (SRT) to determine the peak work rate and to update the individualized cycling workload prescription.

The program combined the following:

(1) HIIT on a cycle ergometer (Lode Corival Home+, Lode BV, Groningen, The Netherlands), enabling remote monitoring. Each HIIT session included 16 intervals of 30 s at 60% of the peak work rate achieved in the most recent SRT, interspersed with 60 s of active recovery at 20 W. Warm-up and cool-down consisted of 3 min at 20 W. Cadence targets were 60–100 rpm during work intervals and 60–80 rpm during warm-up, cool-down, and recovery.

(2) Functional/resistance interval training (15 min), consisting of 30 s work/30 s rest bouts across a circuit of functional exercises (e.g., sit-to-stand, step-ups). Elastic bands and dumbbells were used when needed to progress intensity. Static stretching was recommended at the end of each session.

### 2.6. Measurements

#### 2.6.1. Adherence to the Intervention

Adherence was objectively monitored through the remote tracking system integrated in the cycle ergometer. Compliance with the prescribed training frequency was calculated as the number of completed sessions relative to the three sessions planned per week. Adherence to training intensity was determined by comparing the actual work rate achieved during high-intensity intervals with 60% of the peak work rate recorded in the most recent Steep Ramp Test. Adherence to training duration was evaluated by comparing the effective duration of each session with the target of 25 min. In addition to adherence, the participation rate, reasons for non-participation, drop-out rate, and reasons for drop-out were systematically recorded to assess the overall feasibility of the intervention.

#### 2.6.2. Cardiopulmonary Exercise Test (CPET)

Cardiorespiratory fitness was assessed in an incremental metabolic stress test using a cycle ergometer (Sport Excalibur; Lode, Groningen, The Netherlands) and an oxygen consumption and carbon dioxide production analyzer (K5; Cosmed, Rome, Italy). Before each test, gas analyzers and a flow mass sensor were calibrated using a certified gas mixture and a 3 L syringe, respectively. Before the exercise, a facemask was properly fitted to minimize gas leaks. All parameters were collected breath by breath during one minute at resting condition and 3 min at a warm-up load of 20 W, and a series of 1 W/5 s increments were applied up to voluntary exhaustion. The peak of oxygen consumption (V̇O_2peak_) was obtained in the last 30 s of the test. Absolute (L/min) and relative (mL/kg/min) V̇O_2_, heart rate (bpm), respiratory exchange ratio (ReR) and workload (Watt) were collected at the Gas Exchange Threshold (GET) point, at the Respiratory Compensation Point (RCP) and at the peak of the effort. These variables were defined following the method suggested by Wasserman [28], blindly by two different operators, using a conventional cluster of variables, based on the analysis of trends of the ventilatory equivalent of oxygen (i.e., the ventilation to V̇O_2_ ratio, V̇E/V̇O_2_) and carbon dioxide (V̇E/V̇CO_2_), and end-tidal gas trends. The GET occurs when there is the first acceleration in the increase in ventilation during exercise, which is proportional to the acceleration of the rise in V̇CO_2_ determined by exercise-induced lactic acid production, without a similar increase in V̇O_2_. Above the GET, V̇E/V̇O_2_ rises quickly without a corresponding change in V̇E/V̇CO_2_. As the work rate increases, the pH falls further, and the body responds by stimulating ventilation to increase faster than CO_2_ production. A decrease in end-tidal pressure is observed, reflecting respiratory compensation for the metabolic acidosis induced by exercise. In particular, the RCP, also described as the Respiratory Compensation Point, occurs when there is a further increase in ventilation and in both V̇E/V̇O_2_ and V̇E/V̇CO_2_ ratios [29,30]. The minimum clinically important difference (MCID) score in patients for V̇O_2peak_ is 1.5 mL/kg/min [31].

#### 2.6.3. Steep Ramp Test

The Steep Ramp Test (SRT) was performed at the beginning of each week (during the first training session) to determine the peak work rate, which was then used to individualize the intensity of the subsequent cycling training sessions (see Intervention). SRT was performed in an upright position on a calibrated, electronically braked cycle ergometer (Corival Home+, Lode BV, Groningen, The Netherlands). The Steep Ramp Test begins with a two-minute warm-up involving unloaded cycling. This is followed by an increase in work rate by 10 W every 10 s until voluntary exhaustion is reached. Patients are required to pedal at a frequency of 70–80 revolutions per minute (rpm). The evaluation concludes when participants are unable to maintain 60 rpm despite receiving strong verbal encouragement, after which there is a cool-down period involving unloaded or low-intensity cycling. The peak work rate is defined as the highest wattage reached at the end of the test. The MCID for the SRT is 25–55 W [32].

#### 2.6.4. Knee Extensor Muscle Function

Participants were seated on a fully adjustable, custom-made isometric knee extension device with the knee fixed at 90°. Force was measured via a load cell positioned 2 cm above the malleolus, with the ankle secured to the support using a ratchet strap and a rigid shin guard protecting the tibia. Following progressive warm-up contractions, participants performed two maximal voluntary contractions (MVCs) separated by 2 min of rest, during which they were instructed to push as hard as possible for 5 s with strong standardized verbal encouragement. After an additional 2 min of rest, participants completed 10 explosive contractions interspersed by 15 s of rest, instructed to push as fast and as hard as possible. A visual target corresponding to 80% MVC was provided, and participants were required to exceed this threshold in each contraction. MVC force was calculated as the mean force over a 0.5 s window cantered on peak force. The rate of force development (RFD) was determined from explosive contractions as the peak value of the first derivative of the force signal (20 ms moving average), averaged across the three contractions with the highest RFD. Regarding the MVC, the data available from populations different from the present one found that a change of 31 N or 10% is a reasonable estimate for the MCID [33]. No MCIDs are available for RFD.

#### 2.6.5. Handgrip Strength Test

Handgrip strength (HGS) was measured using a digital dynamometer (Dynx, Akern^®^, Firenze, Italy). The participant is seated with their arm extended alongside their body and is instructed to squeeze the dynamometer with maximal effort, while receiving verbal encouragement. Participants performed three trials with each arm, with 30 s’ rest allowed between trials to minimize fatigue. The highest value obtained across all trials was used in subsequent statistical analyses. The MCID for this test is 5.0 kg [34,35].

#### 2.6.6. 30-Second Sit-to-Stand

The 30-second sit-to-stand test was performed with participants standing in front of a standard chair with feet positioned hip-width apart and arms crossed over their chest with hands on their shoulders. Participants were instructed to perform as many full stands as possible within 30 s. A repetition was only considered valid if the participant clearly made contact with the chair using their thighs or buttocks and then returned to an upright position with knees and hips fully extended. The MCID for 30-second sit-to-stand is ≥2 repetitions [36].

#### 2.6.7. Skeletal Muscle Mass and Quality

Skeletal muscle mass and quality were quantified from routinely acquired abdominal CT scans using a single axial image at the level of the third lumbar vertebra (L3), which is commonly used for opportunistic body composition assessment and correlates with whole-body skeletal muscle estimates [37]. On the selected L3 slice, skeletal muscle compartments (abdominal wall, paraspinal and psoas muscles) were segmented using threshold-based tissue classification; skeletal muscle tissue was identified within a standard attenuation range of −29 to +150 Hounsfield units (HU) [38]. The cross-sectional skeletal muscle area (SMA, cm^2^) was computed as the total area of all muscle pixels, and the skeletal muscle index (SMI) was calculated by normalizing SMA to stature as SMA/height^2^ (cm^2^/m^2^). Skeletal muscle quality was assessed as skeletal muscle radiation attenuation (SMRA), defined as the mean HU of the segmented skeletal muscle area; lower SMRA values reflect greater intramuscular lipid infiltration (myosteatosis) and poorer muscle quality [39].

#### 2.6.8. Questionnaires

Fatigue and health-related quality of life were assessed using validated self-report instruments administered at baseline (pre-intervention) and immediately after completion of the 4-week prehabilitation program (post-intervention). Multidimensional fatigue was measured with the Multidimensional Fatigue Inventory (MFI-20) [40], a 20-item questionnaire comprising five 4-item subscales (General Fatigue, Physical Fatigue, Mental Fatigue, Reduced Activity, and Reduced Motivation), each scored on a 5-point Likert scale; subscale scores range from 4 to 20, with higher scores indicating greater fatigue. Purcell et al. [41] suggested an MCID of 2 points in the MFI-20 in cancer populations.

Health-related quality of life was evaluated using the European Organisation for Research and Treatment of Cancer Quality of Life Questionnaire (EORTC QLQ-C30) [42], a 30-item cancer-specific instrument that yields a Global Health Status/QoL score, five functioning scales, and symptom scales/items; responses are transformed to 0–100 scales according to EORTC scoring procedures, where higher scores represent better functioning/global health and greater symptom burden for symptom scales. When applicable, missing items were handled following the respective scoring manuals (i.e., scale scores were computed only when sufficient item completion was available). The classical MCID for EORTC is quantified as 10 points [43].

Dietary habits were assessed through a food frequency questionnaire, which has been validated in the Italian population [44]

#### 2.6.9. Cytokine/Chemokine Levels

Blood samples were collected into K2EDTA vacutainers and centrifuged. The plasma was collected and stored at −80 °C for subsequent analysis. The Luminex™ 200 system (Luminex, Austin, TX, USA) for multiplexed quantification of human cytokines and chemokines was used. Interleukin (IL)-4, IL-5, IL-8, IL-13, tumor necrosis factor α (TNF-α), CCL11 and CCL27 were simultaneously measured in the samples. All determinations were done in duplicate, and the mean values were used for the analysis. In addition, IL-6 was measured through ELISA (Antibodies.com, Stockholm, Sweden).

### 2.7. Statistical Analysis

Paired pre–post comparisons were conducted using the Wilcoxon test (JASP, Version 0.96.0). When available, MCIDs were adopted to interpret individual changes. Blinding strategies were not applicable by design. No *intention-to-treat* strategy was adopted.

## 3. Results

### 3.1. Feasibility

**Figure 1:**
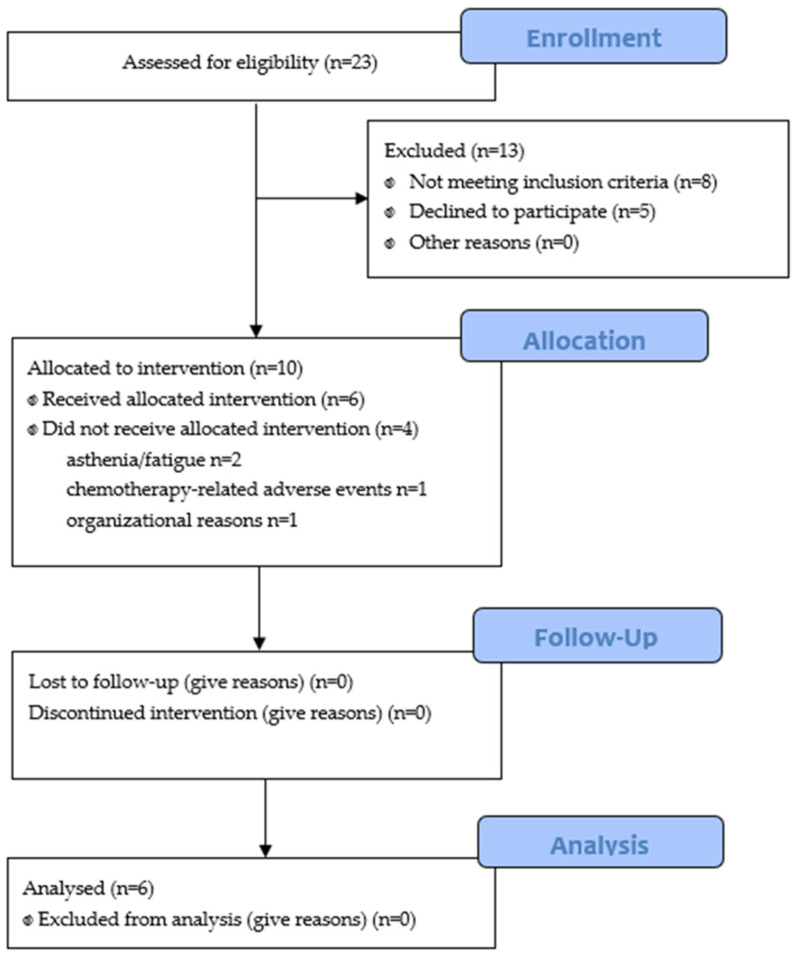
Flowchart of the study.

Patient enrolment was conducted from July 2022 to February 2024. A total of 23 patients were screened, 15 were eligible (65.2%), and 10 were enrolled (66.7% of eligible patients; 43.5% of screened patients) (Figure 1). Four participants (40.0%) discontinued the intervention: two due to asthenia/fatigue, one due to chemotherapy-related adverse events, and one due to organizational reasons. Therefore, six participants (60.0% of enrolled patients) completed the program and the post-intervention assessments (Table 1).

Among completers, compliance with the intervention was 82%, corresponding to a mean of 10 out of 12 scheduled sessions completed. Adherence to training intensity was 100%, as all participants maintained the prescribed intensity corresponding to 60% of the peak work rate during the SRT. Adherence to training duration over the 4-week intervention was 91% overall, with weekly values of 91%, 91%, 89%, and 93% for weeks 1 to 4, respectively. No adverse events occurred during the training sessions.

### 3.2. Clinical Data

All 10 patients enrolled were fit for chemotherapy with a modified FOLFIRINOX chemotherapy regimen with G-CSF support (mFOLFIRINOX: calcium levofolinate 200 mg/m^2^ IV infusion, oxaliplatin 85 mg/m^2^ IV infusion, irinotecan 150 mg/m^2^ IV infusion, fluorouracil 2400 mg/m^2^ IV infusion over 46 h) or PAXG chemotherapy regimen (nab-paclitaxel 150 mg/m^2^, cisplatin 30 mg/m^2^, gemcitabine 800 mg/m^2^, oral capecitabine 1250 mg/m^2^ day 1–28).

All six patients who completed the exercise prehabilitation program received at least 3 months of chemotherapy and underwent surgery after a new evaluation by the MDT. Grade 3 hand–foot syndrome (HFS) was observed in one participant, whereas no other grade 3–4 toxicities were reported (Table 2). Two pancreato-duodenectomies, two distal spleno-pancreatectomies and one total pancreatectomy were performed. In one patient, no resection was performed due to involvement of the mesenteric vessels at surgical exploration. Among the patients undergoing surgery, two encountered postoperative complications (Clavien–Dindo IIIa and IIIb; Table 2). No postoperative mortality occurred.

### 3.3. Secondary Outcomes

**Figure 2:**
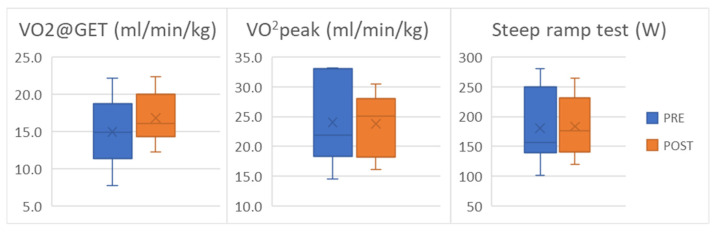
V̇O_2_ at GET (Gas Exchange Threshold), V̇O_2peak_, and maximal power achieved in the Steep Ramp Test are reported before (pre) and after (post) intervention.

No statistically significant improvements were observed in aerobic capacity, as assessed using V̇O_2peak_ and V̇O_2_@GET (Figure 2). At the individual level, three patients improved V̇O_2peak_ beyond the MCID, whereas two patients showed a decrease exceeding the MCID. Additionally, three patients improved their V̇O_2_@GET, and one patient improved performance on the Steep Ramp Test beyond the MCID threshold (Figure S1).

**Figure 3:**
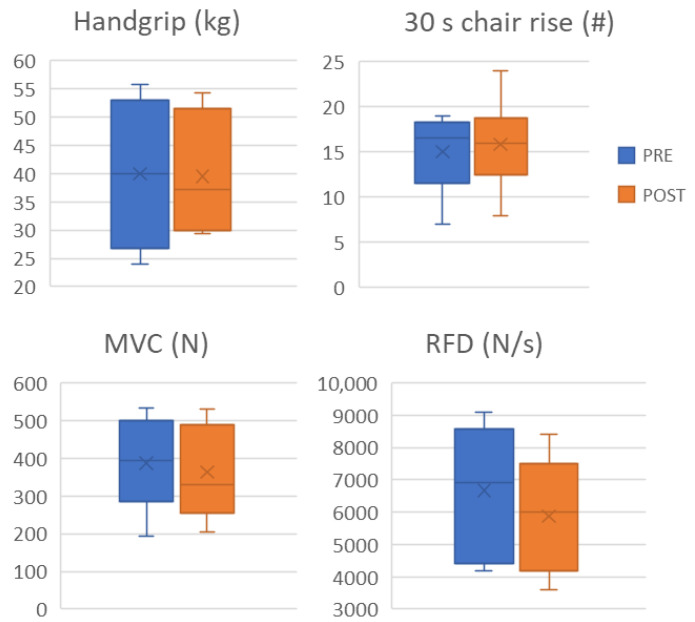
Handgrip strength, 30 s chair rise repetitions, maximal voluntary contraction (MVC) force and rate of force development (RFD) of the knee extensors are reported before (pre) and after (post) intervention.

No statistically significant changes were observed in muscle function, as assessed by handgrip strength, the 30-second sit-to-stand test, MVC force, and knee extensor RFD (Figure 3). At the individual level, one of six patients improved handgrip strength beyond the MCID, and another improved 30-second sit-to-stand performance beyond the MCID threshold. For MVC, one patient demonstrated an improvement exceeding the MCID, whereas another showed a deterioration beyond the MCID (Figure S2).

The skeletal muscle index (SMI) and skeletal muscle radiation attenuation (SMRA) did not change significantly after intervention (Table 3).

**Figure 4:**
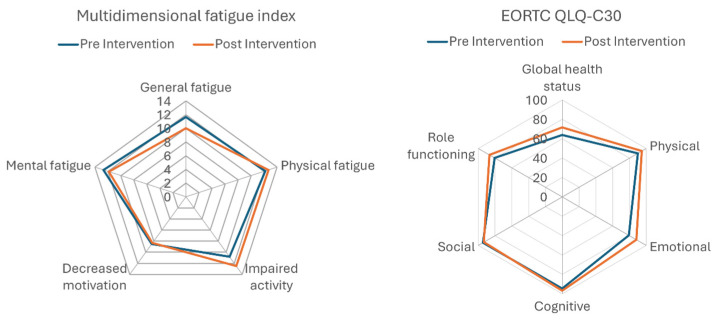
Average scores for multidimensional fatigue index and quality of life before (pre) and after (post) the 4-week prehabilitation program.

No statistically significant differences were observed in either the MFI-20 or EORTC scores before and after the 4-week prehabilitation program (Figure 4). However, at the individual level, two patients demonstrated improvements in EORTC scores exceeding the MCID. For the MFI-20, one patient improved beyond the MCID threshold, whereas one patient exhibited a deterioration exceeding the MCID.

**Figure 5:**
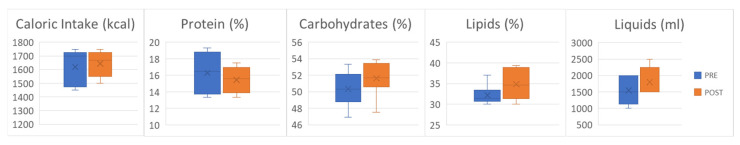
Nutritional parameters (protein, carbohydrates, and lipid are expressed as % of total energy intake) showing pre–post mean, median, and interquartile ranges.

Dietary intakes did not change significantly before–after the intervention (Figure 5). Two patients showed a trend in increasing the percentage of fats.

**Figure 6:**
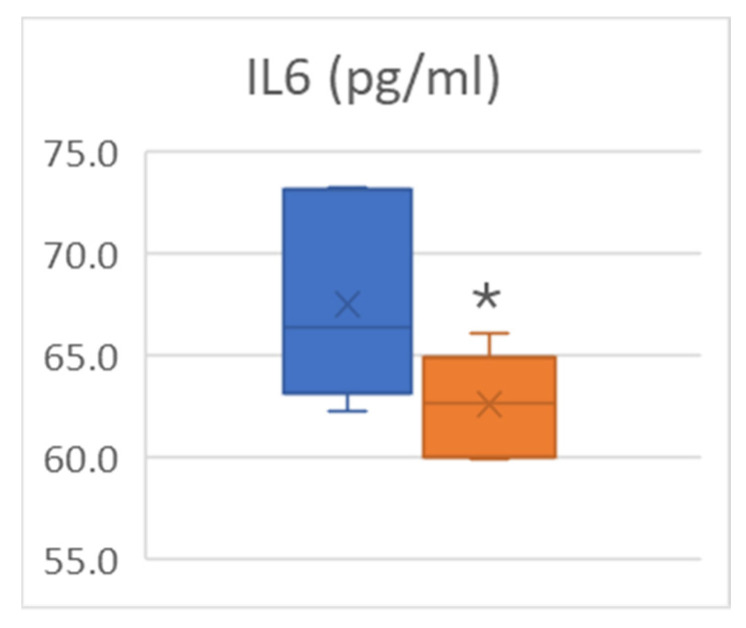
Circulating IL-6 levels before and after intervention. Blue color indicates PRE, orange color indicate POST. * indicates *p* < 0.05.

IL-4, IL-5 and IL-13 serum levels were not in the range of detectability. Other cytokine concentrations were low and did not change after the intervention (Table 4), with the exception of IL-6, whose levels were significantly reduced after intervention (Figure 6).

## 4. Discussion

This pilot feasibility study evaluated a 4-week partially supervised home-based, HIIT-based prehabilitation program implemented during neoadjuvant chemotherapy in pancreatic cancer patients within the available neoadjuvant treatment window. A relevant feature of the intervention was the combination of home-based delivery with objective remote monitoring and partial in-person supervision. Remote monitoring allowed the quantification of adherence and facilitated the early identification of difficulties, while in-person supervision was intended to support safety and individualized progression. The principal findings of the study are as follows: (i) participation was acceptable; (ii) attrition was substantial and was largely driven by fatigue and chemotherapy-related adverse effects; and (iii) among participants who completed the program, cardiorespiratory fitness and muscle function were generally maintained. Although some individuals demonstrated improvements exceeding MCID thresholds, no statistically significant group-level changes were observed in these outcomes. An exploratory reduction in IL-6 was also observed; however, given the single-arm design and the very small completer sample, this finding should be interpreted cautiously.

The lack of significant improvements in physical, functional, and, partially at least, inflammatory outcomes should be interpreted considering the clinical characteristics of the study population. All patients were undergoing neoadjuvant chemotherapy prior to pancreatic surgery. Because patients underwent neoadjuvant chemotherapy before surgical reevaluation, a 4-week prehabilitation intervention was feasible within this treatment window; however, neoadjuvant chemotherapy also introduces a substantial physiological burden including fatigue, sarcopenia, reduced aerobic capacity, and systemic inflammation, in addition to treatment-related adverse events, all of which likely contributed to discontinuation.

The CPET findings indicate that the 4-week intervention did not elicit clear group-level improvements in maximal aerobic capacity, as reflected by V̇O_2peak_. Individual analyses revealed heterogeneous and inconsistent trajectories of aerobic function. Notably, however, V̇O_2_ at the Gas Exchange Threshold improved beyond the MCID in half of the patients. From a physiological standpoint, this pattern is consistent with the adaptations targeted by HIIT, which exposes patients to repeated bouts of high metabolic stress capable of stimulating both central (cardiac output) and peripheral (muscle oxidative capacity, oxygen extraction, lactate handling) mechanisms [45]. Interval work performed at or above the threshold intensity is particularly effective in stabilizing or shifting submaximal aerobic parameters over short training periods, even when changes in peak capacity are modest [46,47]. In the context of neoadjuvant chemotherapy, where rapid deconditioning is expected due to treatment-related fatigue, inactivity, and systemic stress, the maintenance of CPET-derived indices, especially threshold-related V̇O_2_, may be consistent with functional stability during neoadjuvant chemotherapy, although this interpretation remains tentative in the absence of a comparator group.

Across muscle function outcomes (handgrip strength, 30 s chair rise, knee extensor MVC, and RFD), the absence of clear pre–post changes at the group level, together with a limited number of individual trajectories exceeding the prespecified MCIDs, suggests the relative stability of neuromuscular performance in some participants, although preservation cannot be inferred definitively without a control group. This is a relevant signal in the neoadjuvant chemotherapy context, where reductions in habitual activity, treatment-related fatigue, and catabolic/inflammatory stress may otherwise accelerate declines in strength and functional capacity. Importantly, low muscle strength (often operationalized via handgrip strength) and sarcopenia have been associated with a higher risk of major postoperative complications, and lower handgrip strength is also associated with higher mortality risk in cancer and non-cancer populations [48,49]. From a training mechanism standpoint, the functional/resistance component (chair rise/step tasks with external resistance when tolerated) plausibly contributed to maintain task-specific functional performance (chair rise) and general strength (handgrip and MVC); however, the short intervention duration, conservative progression imposed by symptoms, and limited high-velocity loading likely constrained improvements in explosive neuromuscular qualities such as RFD, which are particularly dependent on neural drive and rapid force production stimuli and often require more targeted power-oriented training to change robustly. No improvement was observed in SMI and SMRA over the 4-week period, likely reflecting the short intervention duration, inadequate anabolic support, or the catabolic effects of systemic therapy.

Exercise may modulate inflammatory pathways, and reduced IL-6 has been reported in some exercise-based oncology and non-oncology settings [50,51,52,53]. In accordance, in the present study, IL-6 was the only inflammatory marker showing a statistically significant pre–post change. These data may suggest a possible modulatory effect of the prehabilitation program on the pro-inflammatory milieu. However, this finding should be interpreted with considerable caution, given the very small number of completers, the absence of a comparator group, and the potential influence of multiple biological and treatment-related factors on circulating cytokine levels. Therefore, the observed reduction in IL-6 should be regarded as an exploratory signal rather than evidence of a specific biological effect of the intervention.

With regard to patient-reported outcomes, no statistically significant changes were observed in either EORTC or MFI-20 scores following the 4-week prehabilitation program. In the context of ongoing neoadjuvant chemotherapy—characterized by a substantial symptom burden and treatment-related fatigue—these findings suggest that the intervention did not produce measurable group-level improvements in perceived quality of life or fatigue. Nevertheless, the presence of individual trajectories exceeding the MCID thresholds indicates a heterogeneous response, consistent with the clinical variability of this population, and supports the notion that prehabilitation may at least contribute to stabilizing quality of life and fatigue perception during systemic treatment.

This study has several important limitations typical of early-stage pilot feasibility work. First, only six participants completed the intervention and post-intervention assessments, substantially limiting robustness, precision, and generalizability. Second, the absence of a control group prevents any causal or comparative interpretation of secondary outcome changes. Third, the study population was clinically heterogeneous, including variability in treatment course and chemotherapy regimen, which may have acted as a confounding factor. Fourth, this manuscript reports a center-specific exploratory cohort within a broader multicenter framework, and the findings should therefore not be generalized beyond similar neoadjuvant settings. Finally, several secondary outcomes were exploratory, and the study was not powered to detect clinically meaningful efficacy effects. Accordingly, the present findings should be interpreted primarily in terms of feasibility rather than effectiveness.

## 5. Conclusions

A 4-week partially supervised, home-based exercise prehabilitation program appears feasible in a subset of pancreatic cancer patients undergoing neoadjuvant chemotherapy, with no exercise-related adverse events among those who participated. However, the substantial attrition rate and very small number of completers indicate that feasibility was limited and that implementation remains challenging in this clinical context. Secondary outcomes were largely stable and should be interpreted as exploratory only. Future controlled studies with larger samples are needed to determine whether this approach can be optimized and whether it provides clinically meaningful benefits during neoadjuvant treatment.

## Figures and Tables

**Table 1:** Clinical characteristics of patients concluding the study.

ID	Sex	Age	Weight (kg)	Height (cm)	BMI
001	F	67	70	150	31.1
002	M	47	64	168	22.6
003	M	45	102	184	30.1
004	M	63	82	189	23.0
014	M	61	78	174	25.7
016	M	72	50	150	22.2
Median		62	74	171	24.4
Range		45–72	50–102	150–189	22.2–31.1

**Table 2:** Treatment characteristics of patients concluding the study.

ID	Chemotherapy Regimen	Adverse Events * Chemotherapy-Related	Type of Surgical Resection	Postoperative Complications	pTNM
001	mFFIRINOX	Nausea G1	Pancreato-duodenectomy	Anastomosis ulcer	ypT2; pN1; TRG:4
002	mFFIRINOX	Fatigue G2 Diarrhea G1	Distal spleno-pancreatectomy	None	ypT2; pN0; TRG:3
003	PAX-G	Nausea G1 HFS G1	Exploratory laparotomy: involvement of the mesenteric vessels	N/A	N/A
004	PAX-G	Fatigue G2 Nausea G1 HFS G3 Leukopenia G2	Total pancreatectomy	None	No evidence of residual neoplasia; reactive hyperplasia in lymph nodes (35 findings)
014	mFFIRINOX	Nausea G1	Pancreato-duodenectomy	None	ypT3a; pN0; TRG: 3
016	mFFIRINOX	Nausea G1 Oral mucositis G1	Distal spleno-pancreatectomy	Gastrointestinal bleeding Candida parapsilosis Infection of the central venous catheter	ypT1; pN2; TRG: 4

* Adverse events (AEs) are commonly graded by severity using the Common Terminology Criteria for Adverse Events (CTCAE). N/A not available.

**Table 3:** Skeletal muscle index (SMI) and skeletal muscle radiation attenuation (SMRA) are reported for each patient.

	SMI		SMRA	
ID	PRE	POST	PRE	POST
001	33.2	32.1	34	36
002	32.1	44.9	49	45
003	45.1	48.2	46	50
004	36.5	37.6	53	58
014	38.3	36.1	40	42
016	46.6	46.1	57	62
Median	37.4	41.2	57	62
Range	32.1–46.6	32.1–48.2	34–57	36–62

**Table 4:** Cytokine concentrations are reported for each patient.

ID	CCL27	CCL11	IL-8	TNF-a
01A	73.13	11.67	3.41	1.51
01B	85.36	4.58	2.14	0.67
02A	36.84	5.22	0.51	0.67
02B	48.86	3.83	1.31	0.67
03A	17.09	0.69	0.51	ND
03B	9.34	0.49	0.51	1.51
04A	1.78	0.35	ND	ND
04B	ND	ND	ND	ND
014A	ND	ND	ND	0.67
014B	ND	ND	ND	ND
016A	ND	ND	ND	ND
016B	ND	0.04	ND	ND

## Data Availability

The original contributions presented in this study are included in the article/Supplementary Materials. Further inquiries can be directed to the corresponding author.

## Supplementary Materials

The following supporting information can be downloaded at: https://www.mdpi.com/article/10.3390/medsci14020184/s1, Figure S1. Individual trend of V̇O_2_ at GET (Gas Exchange Threshold), V̇O_2peak_, and maximal power achieved in the steep ramp test are reported before (pre) and after (post) intervention; Figure S2. Individual trend of handgrip strength, 30-s chair rise repetitions, maximal voluntary contraction (MVC) force and rate of force development (RFD) of the knee extensors are reported before (pre) and after (post) intervention.
