# Supplementary material for: Supervised (Home-Based Exercise) Prehabilitation Program in Pancreatic Cancer Patients Undergoing to Neoadjuvant Chemotherapy: A Pilot Feasibility Study
Source: Med Sci (Basel). 2026 Apr 7;14(2):184. doi: 10.3390/medsci14020184 (PMC13108150; doi:10.3390/medsci14020184)

Figure S1. Individual trend of VO_2 at GET (Gas Exchange Threshold), VO_2peak, and maximal power achieved in the steep ramp test are reported before (pre) and after (post) intervention.

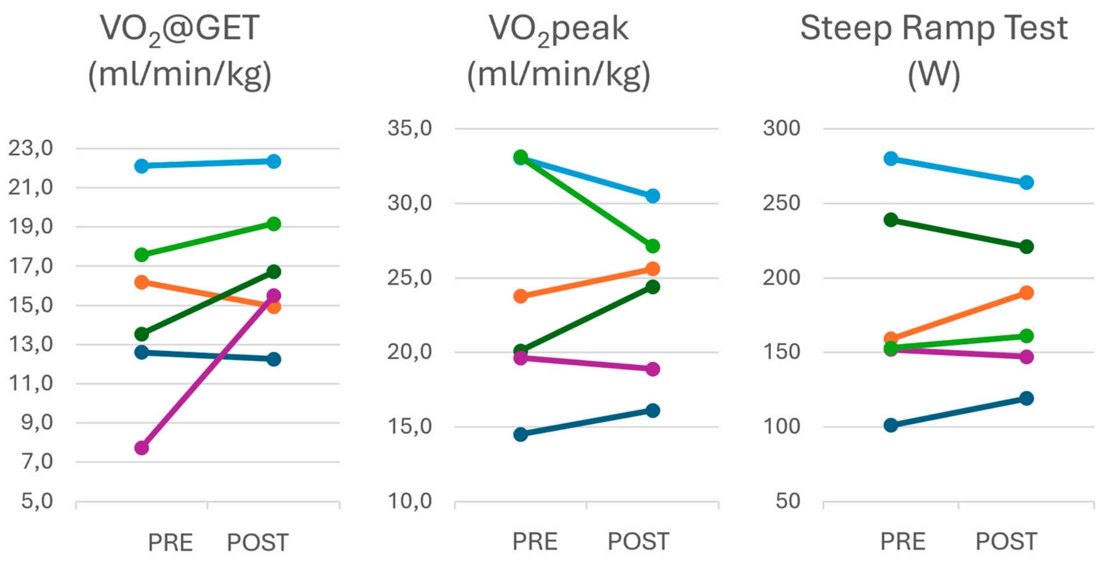

Figure S2. Individual trend of handgrip strength, 30-s chair rise repetitions, maximal voluntary contraction (MVC) force and rate of force development (RFD) of the knee extensors are reported before (pre) and after (post) intervention.

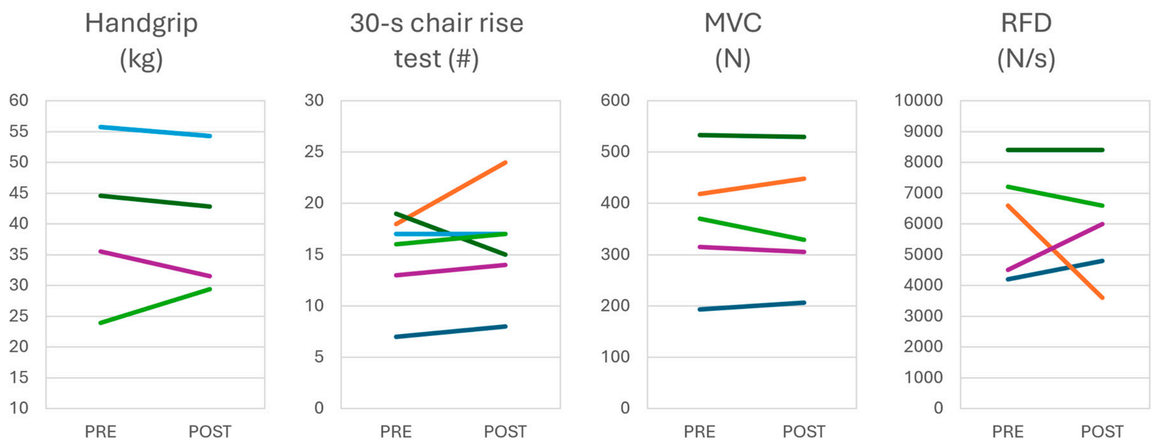

Supplement: Supplementary file 1 [file medsci-14-00184-s001.zip › medsci-4239165-supplementary.pdf]
